# Residual Variation Intolerance Score Detects Loci Under Selection in Neuroinvasive *Listeria monocytogenes*

**DOI:** 10.3389/fmicb.2019.02702

**Published:** 2019-11-26

**Authors:** Bart Ferwerda, Mylène M. Maury, Mathijs C. Brouwer, Lukas Hafner, Arie van der Ende, Stephen Bentley, Marc Lecuit, Diederik van de Beek

**Affiliations:** ^1^Department of Neurology, Amsterdam Neuroscience, Amsterdam UMC, University of Amsterdam, Amsterdam, Netherlands; ^2^Institut Pasteur, Biology of Infection Unit, Inserm U1117 and National Reference Centre – WHO Collaborating Centre for Listeria, Paris, France; ^3^Department of Medical Microbiology, Amsterdam Infection and Immunity, Amsterdam UMC, University of Amsterdam, Amsterdam, Netherlands; ^4^Netherlands Reference Laboratory for Bacterial Meningitis, Amsterdam UMC/RIVM, University of Amsterdam, Amsterdam, Netherlands; ^5^Parasites and Microbes, Wellcome Sanger Institute, Cambridge, United Kingdom; ^6^Paris Descartes University, Division of Infectious Diseases and Tropical Medicine, Necker-Enfants Malades University Hospital, Paris, France

**Keywords:** *Listeria monocytogenes*, neuroinvasive, selection, residual variation intolerance score, genetic variation

## Abstract

*Listeria monocytogenes* is a Gram-positive bacterium that can be found in a broad range of environments, including soil, food, animals, and humans. *L. monocytogenes* can cause a foodborne disease manifesting as sepsis and meningo-encephalitis. To evaluate signals of selection within the core genome of neuroinvasive *L. monocytogenes* strains, we sequenced 122 *L. monocytogenes* strains from cerebrospinal fluid (CSF) of Dutch meningitis patients and performed a genome-wide analysis using Tajima’s D and ω (dN/dS). We also evaluated the residual variation intolerance score (RVIS), a computationally less demanding methodology, to identify loci under selection. Results show that the large genetic distance between the listerial lineages influences the Tajima’s D and ω (dN/dS) outcome. Within genetic lineages we detected signals of selection in 6 of 2327 loci (<1%), which were replicated in an external cohort of 105 listerial CSF isolates from France. Functions of identified loci under selection were within metabolism pathways (*lmo2476*, encoding aldose 1-epimerase), putative antimicrobial resistance mechanisms (*lmo1855*, encoding PBPD3), and virulence factors (*lmo0549*, internalin-like protein; *lmo1482*, encoding comEC). RVIS over the two genetic lineages showed signals of selection in internalin-like proteins loci potentially involved in pathogen-host interaction (*lmo0549*, *lmo0610*, and *lmo1290*). Our results show that RVIS can be used to detect bacterial loci under selection.

## Introduction

*Listeria monocytogenes* is a Gram-positive bacterium adapted to survive in a broad range of environments such as soil, animals, and humans (Radoshevich and Cossart, 2018). Within its natural environment, *L. monocytogenes* is thought to be saprophytic, but when humans ingest this pathogen listeriosis may occur manifesting as sepsis, meningo-encephalitis or maternal-fetal infection (Koopmans et al., 2017; Radoshevich and Cossart, 2018). There are four distinct *L. monocytogenes* lineages of which lineage I has mainly been associated with human disease and lineage II mainly with food contamination (Orsi et al., 2011; Maury et al., 2016; Koopmans et al., 2017). This uneven distribution of lineages may indicate lineages specific environmental pressure with likewise genomic adaptation – beneficial traits that improve an organism’s chance to survive and reproduce which tend to be selected for over time (Vitti et al., 2013). Therefore, detecting genomic regions under selection may suggest underlying biological mechanisms providing insight in the environmental pressure and adaptation history. Such example of *L. monocytogenes* is the selective pressure of commonly used disinfectants, e.g., benzalkonium chloride, in certain populations of strains (Mullapudi et al., 2008; Kremer et al., 2017). In addition, listerial virulence genes and their evolution have been investigated in *L. monocytogenes* populations, e.g., the Listeria Pathogenicity Island 1 containing the *prfA, plcA, hly, mpl, actA*, and *plcB* genes (Tsai et al., 2006, 2011; Orsi et al., 2011; Maury et al., 2016; Hadjilouka et al., 2018). However, less is known about selective pressure of other genes of *L. monocytogenes* core genome.

Bacterial genome wide association studies have been performed to detect regions involved in antibiotic resistance in *Streptococcus pneumoniae*, but other signals of selection are largely unknown (Chewapreecha et al., 2014; Lees et al., 2016). Signatures of selection can be detected by different methodologies, including the residual variation intolerance score (RVIS), ω = dN/dS and Tajima’s D (Tajima, 1989; Kryazhimskiy and Plotkin, 2008; Petrovski et al., 2013). RVIS has been developed within the field of human genetics to evaluate possible pathogenic mutations by evaluating the deviation of amino acid substitutions of a gene compared to the genome average (Petrovski et al., 2013). The score identifies outlier genes that have more (tolerant) or less (intolerant) amino acid substitutions than expected. Many population genetic forces may account for genes that differ from the average number of amino acid substitutions, including selection. Genes that tolerate more amino acid substitutions than the genome wide expectation can therefore indicate either positive or absence of purifying selection. In contrast, genes that are intolerant for amino acid substitutions often reflect purifying selection (Petrovski et al., 2013). The non-synonymous-synonymous ratio, ω = dN/dS, is a method to study selection pressure in bacteria (Petersen et al., 2007; Castillo-Ramirez et al., 2011). This is the ratio between variations altering the amino acid composition and variations that have no implication on the amino acid composition. A ω larger than 1 point to higher number of non-synonymous compared to synonymous variations, indicating positive selection on a locus. Tajima’s D measures the genetic diversity considering the number of pairwise differences and number of segregation sites between isolates (Tajima, 1989).

We applied these different methods on a Dutch cohort of listerial strains isolated from cerebrospinal fluid (CSF) of patients with community-acquired bacterial meningitis (MeninGene) (van de Beek, 2012; Bijlsma et al., 2016). Results of our analysis were validated in a second collection of neuroinvasive *L. monocytogenes* strains cultured from CSF from French patients, providing an overview of genomic regions that may be of importance for interaction between *L. monocytogenes* and its environment.

## Materials and Methods

The Netherlands Reference Laboratory for Bacterial Meningitis (NRLBM) receives bacterial isolates from meningitis patients for national surveillance purposes. All *L. monocytogenes* strains from the NRLBM database and isolated from CSF between 1998 and 2015 were included for analysis. Most of these strains were included in a previous study investigating the strains genetic variation and patient characteristics (Kremer et al., 2017). *L. monocytogenes* was cultured on blood agar plates. DNA extraction was performed using the Wirard^®^ genomic DNA purification kit according to the manufacturer’s protocol (Promega, Madison, WI, United States).

The French National Reference Centre for *Listeria* (NRCL), hosted at Institut Pasteur, provided a matching cohort of 105 *L. monocytogenes* strains from meningitis patients (Supplementary Figure S1). Due to a mandatory declaration system of all listeriosis cases in France, the NRCL receives all *L. monocytogenes* isolates involved in listeriosis throughout the France. To build the replication cohort, we selected isolates involved in neurolisteriosis cases, with a similar number of isolates per serogroup than the NRLBM cohort. Strains from this cohort are referred to as the Pasteur cohort.

Paired end sequencing of 100 nucleotide reads was performed on Illumina HiSeq platform using multiplexed libraries (Illumina, San Diego, CA, United States). Sequences of the 122 isolates were *de novo* assembled using VELVET with default parameters (Zerbino, 2010). *De novo* assemblies were annotated with PROKKA, version 1.11 (Seemann, 2014), and the obtained general feature format (.gff) files were used for further analyzes. The 105-replication *L. monocytogenes* strains from the Pasteur cohort were sequenced and their genomes assembled and annotated using the pipelines of the Pasteur institute, as previously described (Moura et al., 2016).

Roary pan genome pipeline was used to determine and assemble the *L. monocytogenes* core genome of each cohort separately (Page et al., 2015). Different core genomes of each cohort have been constructed using different minimum percentages of identity (i.e., 99% or 85%) and different numbers of isolates that must contain the loci (100% or 15%). Core genomes of isolates from the NRLBM and Pasteur cohorts built with similar parameters were used for replication for each method. To determine overlap of loci between cohorts, open reading frames (ORF) of both core genomes have been annotated according to the BIGSdb-*Lm* database using the batch sequence query tool (Moura et al., 2016). Blast was used to find potential loci matching between cohorts for loci that showed no match after using the BIGSdb-*Lm* database annotation (Altschul et al., 1990).

Residual variation intolerance score is calculated considering the total number of variants within a locus and those that are non-synonymous, i.e., causing amino acid substitutions that could alter the loci function (Petrovski et al., 2013). Roary core alignments consist of genomes made of concatenated loci where variants have been called and annotated, for each strain (Page et al., 2015). Before variant calling and annotation all eight-core genomes were manually checked to avoid any inconsistency within loci containing many repetitive regions, insertions and deletions that could affect the RVIS analysis. SNP-sites were used to extract the variants from all core genomes and to generate a variant call format (VCF) file (Page et al., 2016). Annotation of the variants within the eight VCF files was executed using SnpEff based on each matching core genome template (Cingolani et al., 2012). Number of variants and those that alter the amino acid per locus were determined from the annotated VCF files, which were filtered using vcftools-v0.1.13.4 to exclude all non-biallelic variants and variants with a Minor Allele Frequency (MAF) lower than 0.05 SNPs (Danecek et al., 2011). Regression of all variants on the number of amino acid substitutions was calculated with R using the *lm* function. The studentized residual of the regression was taken as the RVIS and calculated with the *studres* function within the MASS package. Loci with a positive score have more amino acid substitutions than expected and less when the score is negative. It was decided to select the top 1% extremes as interesting loci. The top 1% quartile of the positive scores loci were selected and due to the tolerance of amino acid substitutions will be hereafter referred as tolerant loci. In contrast, the top 1% quartile of the negative scores seem intolerant to amino acid variations and are called the intolerant loci.

The tolerant and intolerant loci could indicate a history of positive or purifying selection. To verify and understand the signals of selection the Tajima’s D and ω were calculated for all loci of the NRLBM isolates. All RVIS, Tajima’s D and ω signals of selection for each method were replicated within the Pasteur cohort loci. Tajima’s D is a measurement of genetic diversity looking at the number of pairwise differences and number of segregation sites between isolates (Tajima, 1989). When no evidence of selection is found in a population for a locus it means that it is evolving per mutation-drift equilibrium resulting in a Tajima’s D = 0. Excess of rare alleles in a population will result in a Tajima’s D < 0 indicating a recent selective sweep. On the other hand, a lack of rare alleles will give a Tajima’s D > 0 which is a sign of balancing selection. In general, loci with values ≤−2 and ≥2 are used as rough measurement of significance. Tajima’s D was calculated using the pegas R package (Paradis, 2010). Excess of non-synonymous (dN) over synonymous (dS) substitutions within a locus could indicate selection. For most loci, the ω = dN/dS tends to be <1. When ω is ≥1 this indicates that the loci could have been subjected to positive selection. It could be that the tolerant loci according to RVIS underwent positive selection resulting in a ω > 1. To test this, ω was estimated for all loci from the 122 NRLBM isolates using CODEML from the PALM 4.9 package and compared to the RVIS results (Yang, 2007). Tajima’s D and ω were calculated for each loci of all isolates and by considering the two lineages separately. Due to lower number of strains per lineage variations were not filtering for MAF. The whole-genome tree was generated by RAxML and used for conditional fitting by CODEML with x = 2 and α = 0 (Stamatakis, 2014).

FastGear was used to determine population genetic structure and to detect recombination for all loci showing a signature of selection (Mostowy et al., 2017). FastGear uses a hidden markov model methodology to identify strain clusters and admixture blocks between them. Admixture blocks within all isolates of the clusters is defined as ancestral recombination. When an admixture block is found only within some isolates of a cluster the recombination is considered recent.

## Results

To compensate for the effect of core genome construction parameters on the methods used to detect signals of selection, results of the four different core genomes were compared based on loci presence and replication success of the RVIS method (22). During core genome construction, specific settings caused the alleles of highly variable loci to be separated into many gene families. This fragmentation was mainly seen when the core locus only had to be present in 15% of the strains. Therefore, all calculations have been performed with loci found in all strains and a minimum percentage identity of 85% (Supplementary Figure S2).

**FIGURE 1:**
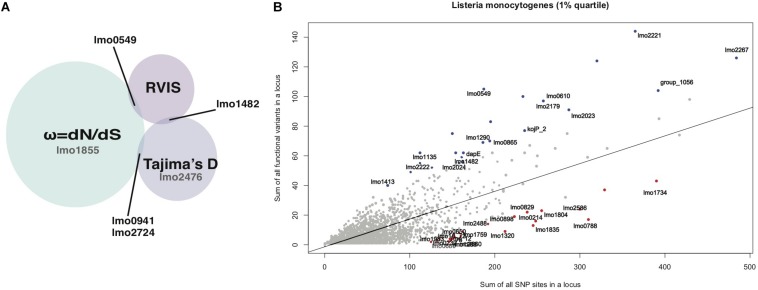
**(A)** Summary of the RVIS, Tajima’s D and ω results of loci that overlap between methods. Loci for which signals of selection were only detected with one method but seen in both lineages are shown in gray. **(B)** Regression plot of the sum of all variants on the sum of all common (>0.05% MAF) non- synonymous substitution for each locus of the NRLBM 85_100 results. The 1% extremes of most intolerant (red) and tolerant (blue) loci replicated between both cohorts have been annotated.

Because the three methods differ in their approach to detect selection and their differential time sensitivity to detect signatures of selection, none of the loci was detected by all methods (Vitti et al., 2013). This overlap does not change when RVIS is conducted on each lineage separately. Overall RVIS depends on a larger accumulation of variation within a locus to detect a signal. In contrast especially ω was detected in small loci because it is less based on the number of variations within a locus. This difference in locus length and detection of the used method is shown in Supplementary Figure S3. Despite the differences of the methods several signals were replicated between the two cohorts and loci were either detected by multiple methods or detected in both lineages (Figure 1A). This resulted in 0.3% (6 out of 2327) of the loci suspected to have a signature of selection.

Residual variation intolerance score analyses were performed on all genomes together, i.e., making no distinction between lineages. Figure 1B shows the regression plot of RVIS results showing the amount of total substitutions found for each locus plotted against the portion of variations leading to amino acid substitutions. Taking the top 1% of tolerant and intolerant loci from the NRLBM cohort resulted in a total of 24 loci for each group. The top 1% was chosen to ensure a sufficient number of loci being included as extreme outliers that most likely underwent selection if the method is capable to detect it in bacterial genomes. Within the 23 loci of the top 1% from the Pasteur cohort, 16 tolerant loci and 19 intolerant loci were replicated between the two cohorts (Figure 1B and Supplementary Table S1). Among the tolerant loci, those encoding internalin-like and LPXTG cell wall anchor proteins were overrepresented (*p*-value = 3.15e-12; fisher exact test; Supplementary Table S1). Non-replicating loci between cohorts were mainly due to absence of the locus within the core genome of one of the cohorts’ strains (see Supplementary Table S2). For the tolerant loci, 6 from the 8 were missing among the genomes of the Pasteur cohort. Higher rate of replication in the intolerant loci was due to the lower genetic variations and 1 out of 5 intolerant loci among the genomes of the NRLBM cohort was not present among those of the Pasteur cohort. No specific pathway or gene function was overrepresented within the intolerant loci.

**FIGURE 2:**
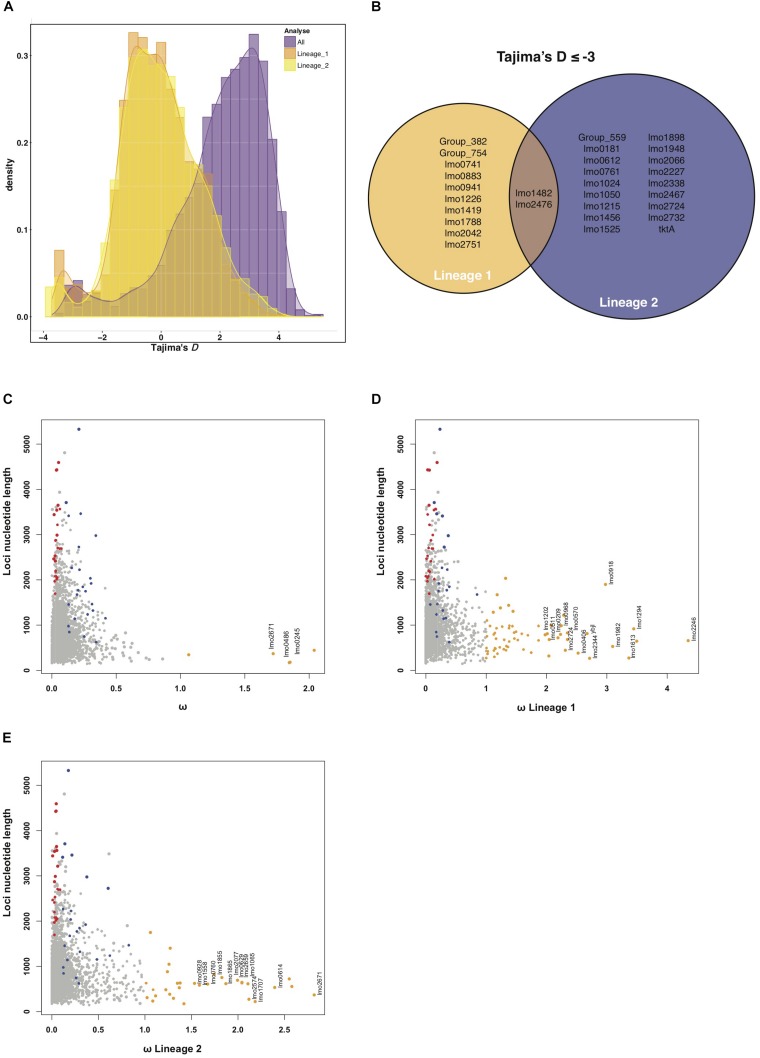
**(A)** Histogram of Tajima’s D results of all strains (purple). What stands out is the shift of Tajima’s D values when all strains are included in the calculation. This shift disappears when Tajima’s D is calculated when strains are grouped by their lineage (orange for lineage I strains and yellow for lineage II strains). All Tajima’s D analysis show an accumulation of loci with values ≤–2.5, indicating positive selection. **(B)** Summarizes the ≤–2.5 loci that were replicated between both the NRLBM and Pasteur cohort. Only two loci show signatures of positive selection within both lineages. **(C)** Nucleotide length plotted against ω for each locus. Loci that show signatures of selection, ω > 1, are colored orange. Only loci of which the signals of selection could be replicated within both cohorts have been annotated. To compare with the RVIS results, all tolerant loci are displayed in blue and intolerant in red. Calculations of ω for lineage I strains only is shown in plot **(D)** where all replicated loci around ω 2 or larger have been annotated. **(E)** Shows lineage II strains only with all ω around 1.5 or larger have been annotated. A complete list of the ω > 1 loci for all and both lineages can be found in Supplementary Table S5.

Calculating Tajima’s D for all genomes of the NRLBM cohort resulted in a shift of the center of the distribution, which was around 2, 59% of the loci having a higher value (Figure 2A). This phenomenon disappeared when Tajima’s D was calculated for each lineage separately. Genetic distance between the two lineages affected the Tajima’s D values tremendously (Figure 2A), which might have a large impact on the interpretation of the results. To avoid the effect caused by the genetic distance between lineages, only the lineage specific Tajima’s D were used.

Distribution of Tajima’s D for both lineages revealed agglomeration of loci with a Tajima’s D ≤ −2.5 (Figure 2A). Twenty-nine loci within this agglomeration were replicated between both cohorts and show signals of positive selection (Figure 2B and Supplementary Table S3). Among lineage I and lineage II genomes, 12 and 20 loci from the 29 showed a signal of selection, respectively. Three loci, *lmo1419* (coding for a hypothetical protein), *lmo1482* (encoding *comEC*) and *lmo2476* (encoding aldose 1-epimerase) showed signals of selection in both lineages.

Signal of positive selection for the *lmo1482* (*comEC*) locus for both lineages was confirmed by RVIS (Figure 1B and Supplementary Table S1). Furthermore, two tolerant loci with RVIS had a Tajima’s D ≤ −2 in lineage I, namely *lmo1290* (encoding internalin-like protein with an LPXTG motif), and *lmo1413* (encoding a putative peptidoglycan bound protein with an LPXTG motif) (Supplementary Table S4). The locus *lmo2023* (*nadB*) in lineage II showed a Tajima’s D ≥ 2, which could indicate balancing selection. The RVIS intolerant loci did not show signals of selection by Tajima’s D.

Calculating ω for all genomes resulted in three loci with a signature of positive selection, namely *lmo0245* (encoding *secE*), *lmo0486* (encoding *rpmF*) and *lmo2671* (coding for a hypothetical protein) (Figure 2C and Supplementary Table S5). Of note, all three loci had a small size, which was less than 500 nucleotides, especially when compared to RVIS which detects selection based on sums of variations resulting in detection within larger loci (Figure 2C and Supplementary Figure S2). Population structure is considered but due the large genetic distance between the lineages ω was also calculated for each lineage separately (Figures 2D,E). For *lmo2671*, selection was also detected when analyzing only lineage II strains. The *lmo1855* locus showed signatures of selection within both lineages (Supplementary Table S5). *lmo1855* encodes a D-alanyl-D-alanine carboxypeptidase also known as PBPD3, which is related to the resistance VanY dd-carboxypeptidase family (Bierne and Cossart, 2007).

Comparing results with RVIS shows that *lmo0549* (encoding an internalin-like protein) was detected by both methods (Supplementary Table S6). Tajima’s D and ω overlapped with *lmo0941* (encoding a hypothetical protein) in lineage I strains and *lmo2724* (encoding a hypothetical protein), for which both methods detected signatures of selection in opposite lineages.

Residual variation intolerance score was also applied to each lineage separately to compare the impact on the results (Supplementary Table S7). Approximately half of the RVIS signals were also detected when applied to the lineage separately. The *lmo0941* locus detected with Tajima’s D and ω was also detected with RVIS but with inconsistency of the lineages.

Population structure and recombination in *L. monocytogenes* could introduce an excess of genetic variation, which can introduce a bias depending on the method used to detect selection. On the other hand recombination could introduce homogeneous regions between strains from different lineages, which could contain important locus or gene regions with essential function for specific environments. The population structure and degree of recombination for the 6 loci that exhibit signatures of selection were investigated with FastGear, which determines the structure of the population using the loci information.

**FIGURE 3:**
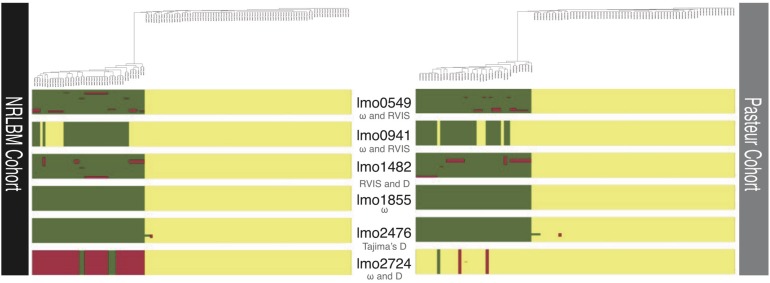
Regions of recombination of the six loci with signatures of selection. Below each locus in gray stands the method which detected the selection for that locus. Above each cohort is the phylogenetic tree of the 122 NRLBM and 105 Pasteur samples. Colors within each locus represent the assigned clusters to each strain and show the ancestral patterns. Similar ancestral colors between strains within the same locus reveal close relatedness and a mixture of ancestral colors marks admixture between strains. The phylogenetic relationships of the strains combined with the ancestral patterns clearly show the strong division of most loci by lineage background of the strains.

The clustering of the strains is thereafter used to determine the genetic similarity between clusters when comparing the genetic regions of the locus. For the six loci that showed signatures of selection, a minimum of 2 and a maximum of 3 different clusters were found (Figure 3), reflecting the lineage background of isolates. When comparing the clusters, small regions of recombination were found for the *lmo0549*, *lmo1482*, and *lmo2476* loci. These regions were mainly found in the strains belonging to lineage II. *lmo1855* showed no evidence of recombination but revealed a clear division between lineages (Figure 3). Within the Pasteur cohort, for loci *lmo0941* and *lmo2724*, similarities according to the lineage genetic background were observed. Overall there was no uniform pattern observed in the six loci under selection.

## Discussion

This study presents six loci within meningitis causing *L. monocytogenes* strains that show signatures of selection using three different methods, namely RVIS, Tajima’s D, and ω. All reported signatures of selection have been verified and replicated within a second independent cohort. With the exception of ω, these methods have not been broadly applied to bacterial genomes because of their high inter-lineages genetic diversity and clonal structure. Within this study, the interpopulation genetic diversity between lineages I and II did not affect the performance of RVIS. Being able to include both lineages during the RVIS analysis gives a different dimension to the detected signatures of selection due to the inclusion of a larger cross section of *L. monocytogenes* strains.

The detected *lmo0549* encodes an internalin-like protein and functional characterization showed that its structure contains WxL domain mediating cell wall binding regions (Bierne and Cossart, 2007; Brinster et al., 2007). Internalin-like proteins are involved in adhesion and invasion of host cells and interact with host cell surface receptors (Schubert et al., 2002; Bonazzi et al., 2009). The function of *lmo0549* is unknown. Coevolution of the *lmo0549* locus with the regulatory sRNAs rli117 is in support of its functional importance (Cerutti et al., 2017). *lmo1855* belongs to the bacterial penicillin-binding proteins and is also known as PBPD3. This locus is related to loci encoding members of the vancomycin resistance VanY dd-carboxypeptidase family but no direct evidence has been reported related to clinical resistance (Bierne and Cossart, 2007; Korsak et al., 2010). Up-regulation of *lmo1855* was seen when growing in mouse spleens and linked to cell wall synthesis (Camejo et al., 2009). The *lmo2476* locus encodes an aldose 1-epimerase, which is involved in the processes of glycolysis (Giotis et al., 2008). *comEC* (*lmo1482*) is part of the DNA uptake competence (Com) system involved in DNA transformation. ComEC is a polytonic membrane protein forming a membrane translocation channel up-regulated during intracellular growth and important in the phagosomal escape of *L. monocytogenes* from macrophages (Rabinovich et al., 2012; Schultze et al., 2015). The function of two hypothetical proteins encoded by *lmo0941* and *lmo2724* is unknown.

Residual variation intolerance score was the only method that could handle with the large genetic distance between the lineages. Among the tolerant loci in RVIS, proteins with an LPXTG motif were frequently detected (Supplementary Table S1). LPXTG motifs are anchored to the cell wall by a sortase (Bierne and Cossart, 2007), and many of the loci with LPXTG motif belong to proteins of the internalins family, some of which are involved in adhesion and invasion of cells by interacting with host cell surface receptors (Schubert et al., 2002; Bonazzi et al., 2009). Signals of selection have been found with RVIS for the cell wall protein genes loci *lmo0549*, *lmo1290* (*inlK*), *lmo1413* (*lmiA*) (Supplementary Table S1). *lmo0549* was also detected by the ω method as well as part of the previously descripted loci. *lmo1290* encodes InlK, which interacts with the Major Vault Protein (MVP), the main component of the cytoplasmic ribonucleoprotein particles, and with this interaction *Listeria* coats its surface with MVP to avoid autophagy (Dortet et al., 2011, 2012; Becavin et al., 2014). *lmo1413* codes for *Listeria*-mucin-binding invasion-A protein (LmiA), interacting with mucin 2 (MUC2) (Mariscotti et al., 2014). Coevolution has been suggested between specific alleles encoding the LmiA and InlA surface proteins (Mariscotti et al., 2014; Radoshevich and Cossart, 2018). Results with RVIS including all genomes of both lineages points toward selection on specific loci, which may be involved in interactions with the host. This difference could reflect the different niches of the two lineages. For a bacterium, which is not thought to cause secondary infection in human, this raises the question of the nature of the environments in which *L. monocytogenes* evolves and where selection takes place. Increasing the number of strains and inclusion of non-clinical strains will be needed to address this question.

Signals of selection must always be interpreted with caution. Our neuroinvasive *L. monocytogenes* lineage I and II isolates are only a small part of the *L. monocytogenes* population.

These cross sections of foodborne *L. monocytogenes* strains had short-term interactions with humans during infection and are not actively transferred between individuals. Due to the short residency and epidemiological dead-end of clinical strains, adaptations shaped by the interaction between bacteria and humans are hard to establish. A example is the *lmo1855* (PBPD3) locus likely involved in β-lactam antibiotics resistance. Selection of the locus could have taken place within soil, cattle, healthcare, or food industry environment making it difficult to associate selection to a specific ecological context (Mathew et al., 2007; Forsberg et al., 2012). Translational studies that link the loci found with specific functional differences can help to resolve underlying forces of selection. The fact that PBPD3 may cause resistance against specific substances may help to deduce the specific events driving the selection.

This study was aimed at testing and comparing different tools to detect signals of selection, and has therefore several limitations. The first limitation is that the analyses were only performed in *L. monocytogenes* isolates causing central nervous system infection. Within lineage I and II, there exists also strains causing other or no disease. Lack of these strains makes it impossible to conclude if the observed signals of selection are related to their pathogenicity. Second, the strains had to be divided by their lineage for the Tajima’s D and ω analysis. In contrast, the RVIS has been conducted on all strains together. The genetic distances between lineages can give a distorted picture of the replication between methods. This has partly been compensated by only selecting those loci that show selection for both lineages when applying Tajima’s D and ω. Third, the presented 6 loci are the result of filter and looking at overlap between used methods. Focus of this study was on the applicability and performance of RVIS for detection signatures of selection. As a result detection of loci under selection by individuals methods is less highlighted. Finally, a signature of selection only makes sense when not only the genetic signal of selection is detected, but the signal of selection has also to be connected with a phenotype that gives insight in the environmental pressure and adaptation providing a functional explanation.

## Conclusion

In conclusion, this study shows that conventional methods (Tajima’s D and ω) could be used to detect signals of selection within *L. monocytogenes*. We used here the RVIS method for the first time on bacterial genomes. RVIS is a fast method that can be used on bacterial strains with a large genetic distance. Therefore, RVIS is a method, that adds to the existing ones to detect signals of selection within bacterial genomes over a longer time period. Overall, signatures of selection were found especially in loci potentially involved in host-pathogen interactions, indicating the importance of understanding the evolutionary past and present.

## Data Availability Statement

All Listeria sequence reads of the Netherlands Reference Laboratory for Bacterial Meningitis (NRLBM) have been deposit in the European Nucleotide Archive (ENA) under project number PRJEB4909 (https://www.ebi.ac.uk/ena/data/view/PRJEB4909). Numbers of the included *Listeria monocytogenes* strains for this study can be found in Supplementary Table S8. NRCL Listeria strains used for replication were specifically composed out of the Listeria collection from ML. The exact strains can be obtained by mailing (marc.lecuit@pasteur.fr.ki).

## Ethics Statement

For the MeninGene study written informed consent was obtained from all patients or their legally authorized representatives. The studies were approved by the Medical Ethics Committee of the Academic Medical Center, Amsterdam, The Netherlands (approval number: NL43784.018.13).

## Author Contributions

BF, MM, and LH contributed to the conception and design of the study. DB, AE, MB, ML, and SB organized the bacterial cohort collection and sequencing. BF performed the analysis and wrote the first draft of the manuscript. BF, MM, LH, DB, and ML discussed the methodology and analysis. All authors contributed to the manuscript revision, read, and approved the submitted version.

## Conflict of Interest

DB received departmental honoraria for serving on a scientific advisory board for GlaxoSmithKline and InflaRx paid to the Academic Medical Center, outside the submitted work. AE declares to have received fees paid to the institution for consultancy activities for GSK and for participation in advisory boards for Pfizer and GSK. SB has received consultancy payments from Merck and Pfizer. The remaining authors declare that the research was conducted in the absence of any commercial or financial relationships that could be construed as a potential conflict of interest.
