# Supplementary material for: Residual Variation Intolerance Score Detects Loci Under Selection in Neuroinvasive Listeria monocytogenes
Source: Front Microbiol. 2019 Nov 26;10:2702. doi: 10.3389/fmicb.2019.02702 (PMC6901971; doi:10.3389/fmicb.2019.02702)
Supplement: Supplementary file 1 [file Data_Sheet_1.PDF]

Supplementary material

Supplementary table 1

RAST[1] Annotation RVIS results separated on tolerant and intolerant.

	RAST Annotation tolerant
dapE	Acetylornithine deacetylase (EC 3.5.1.16)
kojP_2	Maltose phosphorylase (EC 2.4.1.8)
lmo0549	Internalin-like protein Lmo0549 homolog
lmo0610	Internalin-like protein (LPXTG motif)
lmo0865	phosphoglucomutase/phosphomannomutase family protein
lmo1135	FIG00774886: hypothetical protein
lmo1224	ABC transporter%2C permease protein
lmo1290	Internalin-like protein (LPXTG motif)
lmo1413	Putative peptidoglycan bound protein (LPXTG motif)
lmo1482	Late competence protein ComEC%2C DNA transport
lmo2023	L-aspartate oxidase (EC 1.4.3.16)
lmo2024	Quinolinate phosphoribosyltransferase [decarboxylating] (EC 2.4.2.19)
lmo2179	Putative peptidoglycan bound protein (LPXTG motif)
lmo2221	DNA double-strand break repair Rad50 ATPase
lmo2222	DNA double-strand break repair protein Mre11
lmo2267	ATP-dependent nuclease%2C subunit A

	RAST Annotation intolerant
lmo0214	Transcription-repair coupling factor
lmo0232	ATP-dependent Clp protease%2C ATP-binding subunit ClpC / Negative regulator of genetic competence clcC/mecB
lmo0650	phage infection protein
lmo0680	Flagellar biosynthesis protein FlhA
lmo0788	Activator of (R)-2-hydroxyglutaryl-CoA dehydratase
lmo0829	Pyruvate-flavodoxin oxidoreductase (EC 1.2.7.-)
lmo0898	Transcription accessory protein (S1 RNA-binding domain)
lmo1072	Pyruvate carboxyl transferase (EC 6.4.1.1)
lmo1275	DNA topoisomerase I (EC 5.99.1.2)
lmo1286	Topoisomerase IV subunit B (EC 5.99.1.-)
lmo1320	DNA polymerase III polC-type (EC 2.7.7.7)
lmo1734	Glutamate synthase [NADPH] large chain (EC 1.4.1.13)
lmo1759	ATP-dependent DNA helicase UvrD/PcrA
lmo1804	Chromosome partition protein smc
lmo1835	Carbamoyl-phosphate synthase large chain (EC 6.3.5.5)
lmo1983	Dihydroxy-acid dehydratase (EC 4.2.1.9)
lmo2488	Excinuclease ABC subunit A
lmo2586	Formate dehydrogenase related protein
lmo2660	Transketolase (EC 2.2.1.1)

1 Overbeek, R. et al. The SEED and the Rapid Annotation of microbial genomes using Subsystems Technology (RAST). Nucleic Acids Res 42, D206-214, doi:10.1093/nar/gkt1226 (2014).

Supplementary table 2

All results that did not replicate between NRLBM and Pasteur cohorts

	Tolerant			Intolerant	
	NRLBM	Pasteur		NRLBM	Pasteur
lmo0204	✔	-	lmo0007	✔	+
lmo0323	-	✔	lmo0841	+	✔
lmo0333	✔	-	lmo1641	✔	+
lmo0411	+	✔	lmo1644	-	✔
lmo0438	✔	+	lmo1811	✔	+
lmo0576	✔	-	lmo1923	+	✔
lmo0789	-	✔	lmo2378	✔	+
lmo0939	✔	-	lmo2679	✔	-
lmo0941	✔	+	lmo2681	+	✔
lmo1129	-	✔			
lmo1136	✔	-			
lmo1910	+	✔			
lmo2015	+	✔			
lmo2821	✔	-			

✔ Within RVIS 1% quartile
+ Locus present in core genome
- Locus absent in core genome

Supplementary table 3

Replication of Tajima's D outcome loci ≤ -2.5 for lineage I and lineage II

	Lineage I		Lineage II	
Locus	Tajima's D NRLBM	Tajima's D Pasteur	Tajima's D NRLBM	Tajima's D Pasteur
lmo0085	-3.467	-3.162	-3.668	-
lmo0671	-3.427	-3.455	-	-
lmo0741	-3.056	-3.403	-	-
lmo0883	-3.344	-3.390	-	-
lmo0941	-3.334	-3.488	-	-
lmo1226	-3.469	-3.536	-	-
lmo1419	-3.465	-3.507	-2.933	-3.726
Lmo1482	-3.433	-3.380	-3.380	-2.998
Lmo1788	-3.436	-3.507	-	-
Lmo2042	-3.265	-3.329	-	-
Lmo2476	-3.205	-3.500	-3.004	-3.143
Lmo2751	-3.355	-3.330	-	-
Lmo2740	-	-	-3.219	-3.200
Lmo0181	-3.139	-	-3.603	-3.760
Lmo0612	-3.105	-	-3.577	-3.641
Lmo0761	-	-	-3.375	-3.536
Lmo1024	-	-3.499	-3.318	-3.447
Lmo1050	-	-	-3.412	-3.645
Lmo1215	-	-	-3.255	-3.343
Lmo1456	-	-	-3.319	-3.325
Lmo1525	-	-	-3.087	-3.167
Lmo1898	-	-	-3.461	-3.453
Lmo1948	-	-	-3.693	-3.562
Lmo2066	-	-	-3.532	-3.584
Lmo2227	-	-	-3.332	-3.774
Lmo2338	-	-	-3.590	-3.633
Lmo2724	-	-	-3.240	-3.558
Lmo2732	-	-	-3.160	-3.295
tktA	-	-	-3.214	-3.241

Supplementary table 4

Tajima's D results for replicated RVIS tolerant and intolerant results

RVIS tolerant results

	Tajima's D NRLBM			Tajima's D Pasteur		
	All	L1[a]	L2[b]	All	L1[a]	L2[b]
dapE	4.000	0.277	0.818	3.632	0.112	-0.985
kojP_2	3.927	1.570	-0.328	3.694	1.110	0.372
lmo0549	2.680	-1.775	1.210	-2.365	-3.299	-3.492
lmo0610	4.097	-0.359	0.192	-2.842	-3.508	-1.993
lmo0865	2.366	-0.220	0.713	2.093	0.154	1.024
lmo1135	1.470	-0.236	1.553	2.074	2.194	0.997
lmo1224	3.614	1.288	-0.255	3.227	0.634	-0.837
lmo1290	2.394	-2.035	0.871	-2.508	-3.510	-0.521
lmo1413	1.480	-2.101	0.110	-2.939	-3.479	-0.620
lmo1482	-3.130	-3.433	-3.380	-1.754	-3.380	-2.996
lmo2023	0.718	1.527	2.338	0.928	0.140	2.610
lmo2024	-0.108	-1.113	1.846	-0.276	-0.851	1.542
lmo2179	-2.826	-0.511	-3.659	2.638	-1.951	0.073
lmo2221	4.468	-0.146	-0.503	4.251	-0.375	-1.229
lmo2222	4.019	-0.750	0.287	3.858	-0.534	-0.279
lmo2267	4.247	-0.870	0.541	-2.663	-3.525	-3.603

RVIS intolerant results

	Tajima's D NRLBM			Tajima's D Pasteur		
	All	L1[a]	L2[b]	All	L1[a]	L2[b]
lmo0214	2.972	-0.543	0.834	2.621	-0.944	0.769
lmo0232	4.142	1.201	-0.687	4.029	-0.264	-0.212
lmo0650	3.367	0.336	-0.065	3.405	1.842	-0.003
lmo0680	2.897	-0.676	0.122	3.405	1.842	-0.003
lmo0788	-2.578	-3.270	0.286	3.405	1.842	-0.003
lmo0829	1.905	0.480	0.985	1.603	1.765	0.305
lmo0898	3.495	1.752	-0.812	3.766	1.166	-0.995
lmo1072	2.973	0.350	-1.495	-3.208	-3.523	0.088
lmo1275	3.821	0.314	-1.370	3.437	0.127	0.545
lmo1286	3.408	2.409	1.023	3.096	1.498	2.010
lmo1320	1.648	-0.526	0.850	1.689	-0.099	1.329
lmo1734	-3.063	-0.045	-3.360	1.318	0.645	1.895
lmo1759	3.219	1.203	0.323	2.846	1.706	1.151
lmo1804	2.548	0.437	-0.628	2.280	-0.141	-2.371
lmo1835	4.217	-0.432	0.281	3.571	-0.504	-2.386
lmo1983	2.519	-1.029	1.425	2.346	-1.085	1.497
lmo2488	3.198	0.445	1.213	3.178	0.521	0.501
lmo2586	1.401	0.926	1.811	1.301	0.354	1.875
lmo2660	3.880	-0.782	0.123	3.625	-1.464	-0.832

[a] Lineage 1
[b] Lineage 2

Supplementary table 5

Replication of ω outcome loci ≥ 1 for all, lineage I and lineage II

	All		Lineage I		Lineage II	
Locus	ω NRLBM	ω Pasteur	ω NRLBM	ω Pasteur	ω NRLBM	ω Pasteur
immR_1	-	-	1.225	1.108	-	-
lmo0083	-	-	1.188	1.388	-	-
lmo0095	-	-	-	-	1.378	1.116
lmo0155	-	-	1.590	1.764	-	-
lmo0190	-	-	-	-	1.242	1.276
lmo0209	-	-	2.190	9.937	-	-
lmo0230	-	-	1.360	1.140	-	-
lmo0245	1.852	1.850	-	-	-	-
lmo0397	-	-	-	-	1.225	1.680
lmo0398	-	-	1.618	6.496	-	-
lmo0406	-	-	2.524	2.264	-	-
lmo0486	1.846	1.134	-	-	-	-
lmo0499	-	-	1.523	1.470	-	-
lmo0511	-	-	2.045	1.585	-	-
lmo0549	-	-	1.322	1.469	-	-
lmo0570	-	-	2.346	1.894	-	-
lmo0614	-	-	-	-	2.395	1.860
lmo0629	-	-	-	-	1.369	1.359
lmo0658	-	-	-	-	2.042	1.301
lmo0760	-	-	-	-	1.674	1.814
lmo0777	-	-	-	-	1.266	1.216
lmo0845	-	-	1.012	1.037	-	-
lmo0918	-	-	2.977	6.250	-	-
lmo0928	-	-	-	-	1.533	1.512
lmo0941	-	-	1.867	1.636	-	-
lmo0968	-	-	2.232	8.933	-	-
lmo0975	-	-	1.298	7.455	-	-
lmo1065	-	-	-	-	2.107	1.487
lmo1202	-	-	1.977	1.284	-	-
lmo1281	-	-	1.025	10.581	-	-
lmo1294	-	-	3.446	2.380	-	-
lmo1558	-	-	-	-	1.585	1.962
lmo1613	-	-	3.365	3.081	-	-
lmo1707	-	-	-	-	2.185	2.617
lmo1832	-	-	1.466	1.121	-	-
lmo1855	-	-	1.143	2.965	1.744	1.551
lmo1865	-	-	-	-	1.869	3.707
lmo1935	-	-	1.062	1.128	-	-
lmo1940	-	-	1.265	3.310	-	-
lmo1982	-	-	3.096	3.804	-	-
lmo2077	-	-	-	-	1.995	1.160
lmo2104	-	-	-	-	1.419	4.318
lmo2246	-	-	4.348	21.679	-	-
lmo2253	-	-	-	-	1.012	2.759
lmo2344	-	-	2.714	3.587	-	-
lmo2431	-	-	1.434	1.002	-	-
lmo2439	-	-	1.746	2.448	-	-
lmo2516	-	-	1.382	1.728	-	-
lmo2574	-	-	-	-	2.117	5.722
lmo2579	-	-	-	-	1.306	7.075
lmo2597	-	-	1.128	2.059	-	-
lmo2659	-	-	-	-	2.042	9.397
lmo2671	1.721	1.217	-	-	2.819	1.403
lmo2724	-	-	2.312	1.376	-	-
ybjI	-	-	2.672	7.749	-	-

Supplementary table 6

ω results for replicated RVIS tolerant and intolerant results

RVIS tolerant results

	ω NRLBM			ω Pasteur		
	All	L1[a]	L2[b]	All	L1[a]	L2[b]
dapE	0.178	0.297	0.166	0.199	0.325	0.190
kojP_2	0.154	0.268	0.121	0.141	0.440	0.085
lmo0549	0.299	1.322	0.202	0.256	1.615	0.211
lmo0610	0.205	0.346	0.268	0.222	0.685	0.264
lmo0865	0.195	0.848	0.192	0.197	1.734	0.192
lmo1135	0.416	0.333	0.484	0.432	0.329	0.494
lmo1224	0.131	0.274	0.115	0.131	0.237	0.132
lmo1290	0.241	0.390	0.297	0.231	0.444	0.218
lmo1413	0.319	0.281	0.301	0.298	0.191	0.278
lmo1482	0.215	0.355	0.206	0.197	0.186	0.194
lmo2023	0.131	0.073	0.137	0.139	0.100	0.131
lmo2024	0.136	0.172	0.125	0.151	0.152	0.131
lmo2179	0.224	0.180	0.215	0.220	0.123	0.264
lmo2221	0.210	0.304	0.605	0.214	0.306	0.518
lmo2222	0.277	0.176	0.622	0.286	0.116	0.492
lmo2267	0.111	0.138	0.139	0.120	0.109	0.167

RVIS intolerant results

	ω NRLBM			ω Pasteur		
	All	L1[a]	L2[b]	All	L1[a]	L2[b]
lmo0214	0.039	0.132	0.028	0.032	0.096	0.026
lmo0232	0.015	0.011	0.008	0.014	0.021	0.001
lmo0650	0.074	0.115	0.087	0.083	0.089	0.088
lmo0680	0.032	0.001	0.047	0.032	0.001	0.055
lmo0788	0.037	0.030	0.045	0.032	0.026	0.040
lmo0829	0.048	0.053	0.050	0.046	0.049	0.044
lmo0898	0.029	0.063	0.036	0.029	0.057	0.032
lmo1072	0.017	0.030	0.008	0.015	0.030	0.001
lmo1275	0.027	0.041	0.032	0.025	0.029	0.027
lmo1286	0.022	0.025	0.026	0.022	0.025	0.019
lmo1320	0.035	0.058	0.041	0.036	0.113	0.029
lmo1734	0.052	0.186	0.047	0.054	0.221	0.051
lmo1759	0.030	0.047	0.028	0.031	0.043	0.030
lmo1804	0.062	0.160	0.534	0.071	0.122	0.062
lmo1835	0.043	0.060	0.062	0.045	0.087	0.041
lmo1983	0.026	0.056	0.027	0.022	0.001	0.032
lmo2488	0.030	0.084	0.028	0.030	0.045	0.022
lmo2586	0.040	0.113	0.035	0.041	0.110	0.039
lmo2660	0.038	0.139	0.443	0.041	0.111	0.054

[a] Lineage 1
[b] Lineage 2

Supplementary table 7

RVIS results when applied to each lineage separately.

RVIS Tolerant results				RVIS Intolerant results			
Locus	RVIS Lineage I	Locus	RVIS Lineage II	Locus	RVIS Lineage I	Locus	RVIS Lineage II
kojP_2	✓[1]	dapE	p[1]	lmo0237	n	lmo0123	p
lmo0017	p[1]	lmo0204	n*	lmo0259	✓	lmo0214	✓[1]
lmo0075	p	lmo0270	✓	lmo0519	n	lmo0574	p
lmo0297	n	lmo0411	✓	lmo0788	✓[1]	lmo0680	n[1]
lmo0323	p*	lmo0429	n	lmo0813	✓	lmo0707	n
lmo0333	n*	lmo0439	✓	lmo0826	n*	lmo0788	✓
lmo0434	n*	lmo0588	✓	lmo0829	✓[1]	lmo0818	n
lmo0438	✓	lmo0789	p*	lmo1096	p*	lmo0837	✓
lmo0528	✓	lmo0824	n	lmo1238	p*	lmo0841	✓
lmo0540	p	lmo0865	✓[1]	lmo1286	n[1]	lmo1156	n
lmo0601	✓	lmo0939	n*	lmo1325	✓	lmo1234	P
lmo0764	✓	lmo0941	✓[2]	lmo1634	n	lmo1255	✓
lmo0865	p[1]	lmo1258	✓	lmo1641	p	lmo1331	p*
lmo1128	p*	lmo1729	✓	lmo1661	p	lmo1472	p
lmo1129	✓	lmo1798	✓	lmo1718	✓	lmo1537	n
lmo1132	✓	lmo1867	✓	lmo2019	✓	lmo1538	n
lmo1135	✓[1]	lmo2014	✓	lmo2092	n*	lmo1644	p*
lmo1136	n*	lmo2015	p	lmo2145	n	lmo1664	✓
lmo1374	n	lmo2102	n	lmo2372	n	lmo1734	n[1]
lmo1790	✓	lmo2221	✓[1]	lmo2404	✓	lmo1759	✓[1]
lmo1867	n	lmo2222	✓[1]	lmo2411	p	lmo1768	✓
lmo2014	n	lmo2781	✓	lmo2419	✓	lmo1804	n[1]
lmo2046	✓	lmo2812	✓	lmo2547	✓	lmo1983	✓[1]
lmo2224	n*	lmo2820	✓	lmo2664	p	lmo2377	✓
lmo2688	✓	lmo2821	n*	lmo2687	✓	lmo2378	✓
lmo2689	✓			lmo2810	✓	lmo2586	✓[1]
lmo2755	✓			lmo2842	n	lmo2758	N
				rho	✓	lmo2810	✓
						lmo2811	✓
						lmo2818	p
						lmo2826	p

✓ = RVIS tolerant, replicated
n = RVIS within NRLBM cohort only
p = RVIS within Pasteur cohort only
* = Locus only found within this cohort
1 = Also found with RVIS
2 = Also found signal with ω and Tajima's D

Supplementary figure 1

Comparison of the 122 NRLBM strains with the 105 replication strains from the Pasteur cohort. A) Displays the midpoint rooted phylogenetic tree constructed with RAxML, using the core genomes, of each cohort separately. Comparing the trees show that about same numbers of strains are divided between lineage I and II. With 79 lineage I and 43 lineage II strains included in the NRLBM and 67 lineage I and 38 lineage II strains within the Pasteur cohort. More genetic diversity is observed in strains from lineage II. B) A new core genome was constructed with strains from both cohorts and RAxML was used to reconstruct the phylogenetic tree. The midpoint rooted phylogenetic tree is accompanied by information of a strains lineage, serotype and cohort. Overall no dominant clustering is observed between strains from one cohort.

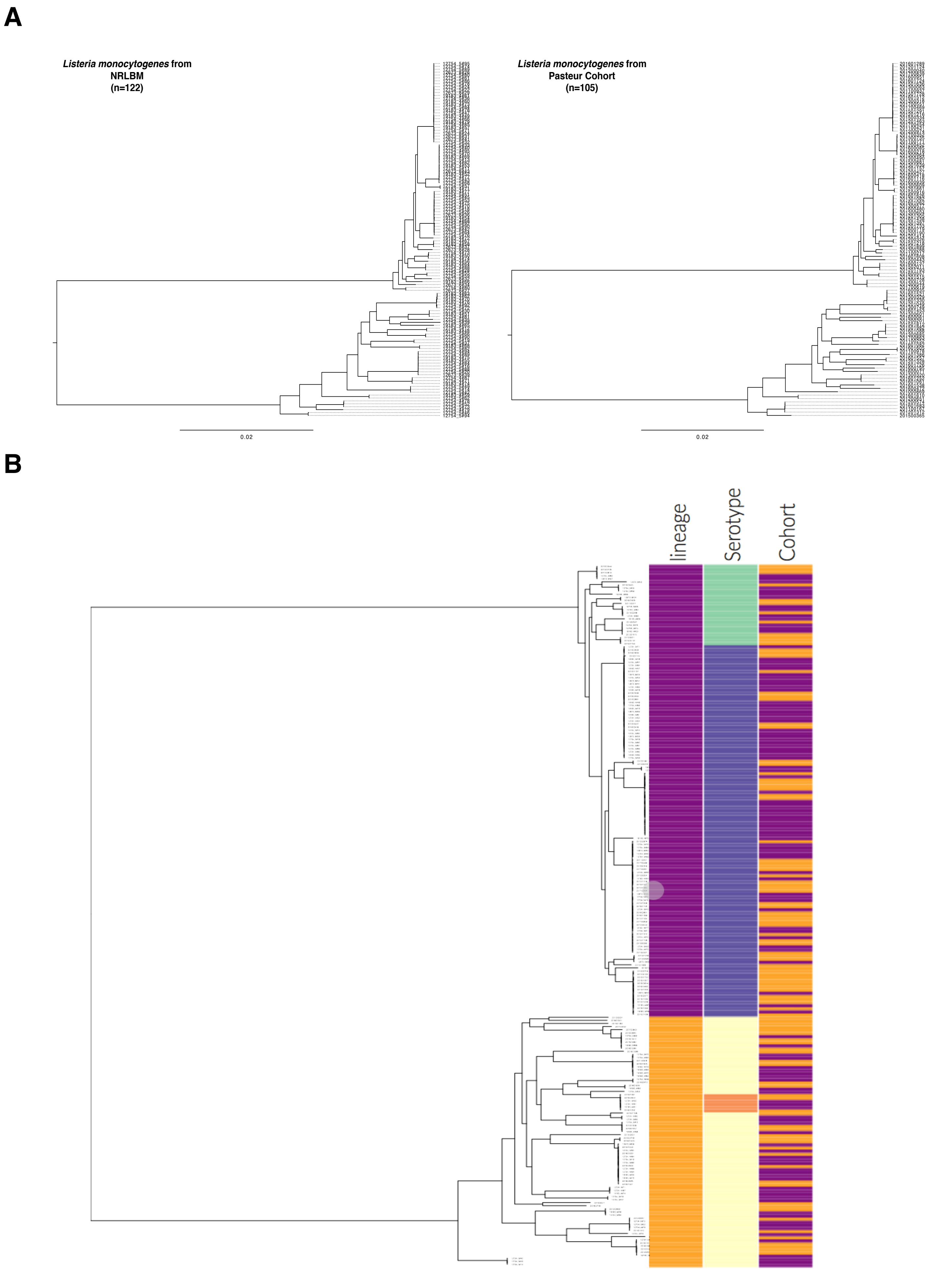
A
Listeria monocytogenes from
NRLBM
(n=122)
Listeria monocytogenes from
Pasteur Cohort
(n=105)
0.02
0.02
B
lineage
Serotype
Cohort

Supplementary figure 2

Figure 2 A) shows the overlap between NRLBM and Pasteur cohort loci and RVIS results grouped on tolerant and intolerant loci. Depending on the roary settings a minimal of 1268 and maximum of 3185 loci were determined for the NRLBM cohort and 1261-4278 loci the Pasteur cohort. When setting the parameters to 85% locus similarity and 15% of the strains containing the locus showed a 2.5-3.4-fold increase in loci. Despite the increase in number of loci this is associated with an increase in the locus fragmentation. Fragmentation is when a locus has high genetic variation and therefore is divided as several different loci during the core genome composition. The fragmentation is shown in the pie chart above each group. Same dynamics were found in both cohorts.

Similarity of called loci between the NRLBM and Pasteur cohorts and replication success of the RVIS method is shown in figure 2B. Blue circled + are the RVIS tolerant loci and the intolerant loci are shown as the red circled -. Below both tolerant and intolerant groups the replication percentages are displayed. The middle shows the roary settings of the data set. A locus similarity of 85% and all strains must contain it showed the best replication.

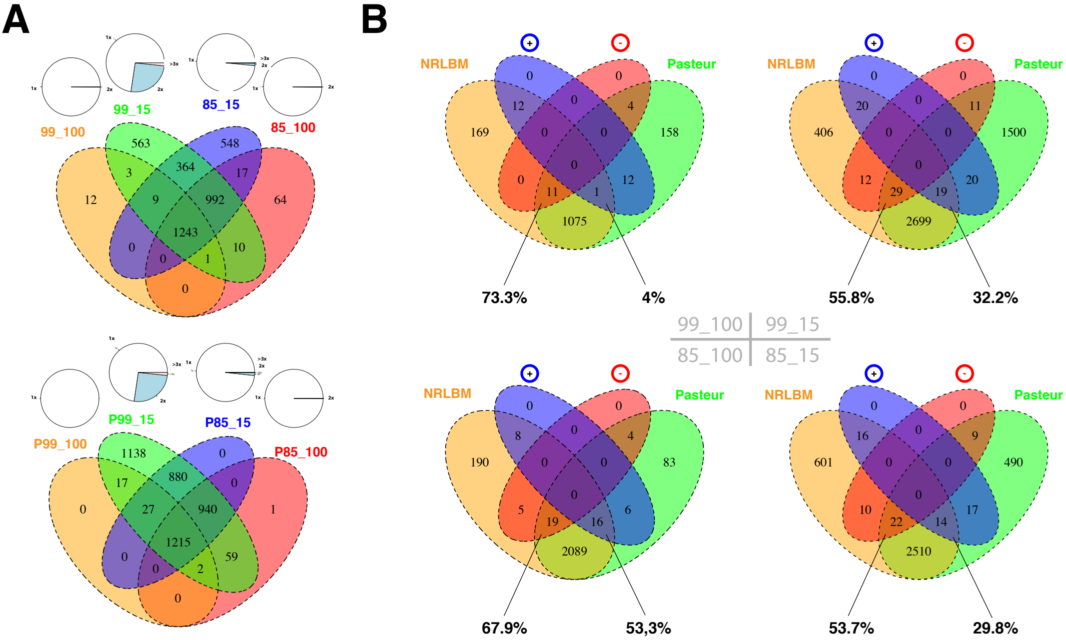

A
B
99_15
85_15
99_100
85_100
563
548
3
364
17
12
9
992
64
1243
0
10
0
1
0
P99_15
P85_15
P99_100
P85_100
1138
0
17
880
0
0
27
940
1
1215
0
59
0
2
0
NRLBM
Pasteur
+
-
0
0
12
0
4
169
0
0
158
0
0
12
11
1
1075
73.3%
4%
20
0
11
406
0
0
1500
0
12
20
29
19
2699
55.8%
32.2%
99_100
99_15
85_100
85_15
8
0
4
190
0
0
83
0
5
6
19
16
2089
67.9%
53,3%
16
0
9
601
0
0
490
0
10
17
22
14
2510
53.7%
29.8%

Supplementary figure 3

Overview length of the replicated loci for the three different methods is given in panel A. RVIS is separated on the loci detected as tolerant (blue) and intolerant (red). Lineages for Tajima's D and ω (dN/dS) are indicated with yellow for lineage I, orange for lineage II and black when the signal was detected within all strains. B) shows the RVIS score of all loci with there corresponding loci length. Colour of the loci indicates when selection of a locus was selected with on of the used methods. (RVIS tolerant is indicated by blue and intolerant with red, Tajima's D with purple or ω (dN/dS) with pink). Special interest is the small locus size of the loci detected with ω in contrast to the other methods.

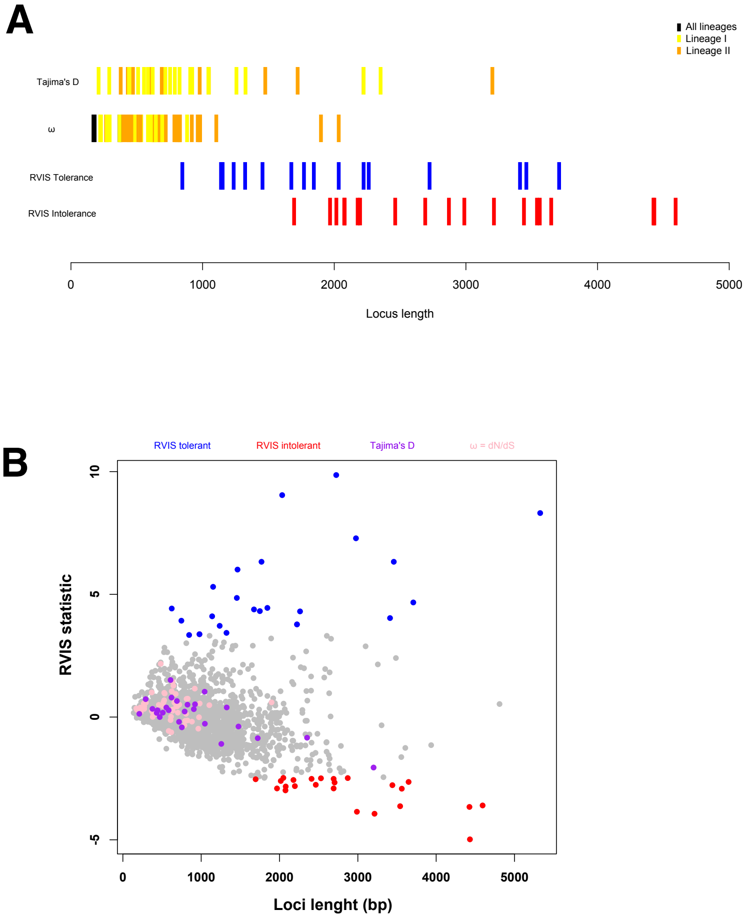

A
All lineages
Lineage I
Lineage II
Tajima's D
ω
RVIS Tolerance
RVIS Intolerance
0
1000
2000
3000
4000
5000
Locus length
B
RVIS tolerant
RVIS intolerant
Tajima's D
ω = dN/dS
RVIS statistic
10
5
0
-5
0
1000
2000
3000
4000
5000
Loci lenght (bp)
